# Effect of “Spatially Confined” Sepiolite on the Processing and Properties of Natural Rubber/Silica Composites Prepared by Latex Compounding Method

**DOI:** 10.3390/polym18080962

**Published:** 2026-04-15

**Authors:** Zhanfeng Hou, Yahe Hou, Qi Chen, Hui Yang, Hongzhen Wang, Zhenxiang Xin

**Affiliations:** 1 School of Materials Engineering, Xuzhou College of Industrial Technology, Xuzhou 221140, China; houyh1357@163.com (Y.H.);; 2 Key Laboratory of Rubber-Plastics, Ministry of Education, Shandong Provincial Key Laboratory of Rubber-Plastics, School of Polymer Science and Engineering, Qingdao University of Science and Technology, Qingdao 266042, Chinaqustwhz@163.com (H.W.)

**Keywords:** natural rubber (NR), sepiolite, silica, latex compounding, spatial confinement, filler loss

## Abstract

To address the pronounced self-aggregation of highly loaded silica in the aqueous phase and the substantial filler loss occurring during the flocculation stage of latex compounding, this study introduces disaggregated and activated sepiolite possessing a spatial confinement effect as both a suspension stabilizer and a synergistic reinforcing component. On this basis, a multiscale natural rubber (NR)/silica/sepiolite composite system was constructed via a latex compounding route. Rheological characterization combined with static sedimentation observations revealed that the percolation threshold of the sepiolite is approximately 0.8 wt%. When the sepiolite content exceeds 1.0 wt%, its fibrous morphology enables the formation of a continuous three-dimensional network, which physically constrains silica particles and effectively suppresses their sedimentation and self-aggregation in the aqueous medium. Guided by this percolation behavior, a stable silica/sepiolite hybrid slurry was subsequently wet-mixed with natural rubber latex, and the influence of sepiolite loading on silica retention during flocculation, as well as on the resulting composite properties, was systematically examined. The results demonstrate that incorporation of sepiolite reduces filler loss during flocculation, with the loss rate decreasing from 4.7% to 1.1%. The Payne effect, SEM, dynamic and static mechanical analyses indicate that an appropriate sepiolite dosage promotes dispersion of silica within the rubber matrix while simultaneously strengthening filler–rubber interfacial interactions. Accordingly, tensile and tear strengths are increased from 32.1 to 35.5 MPa and from 92.3 to 133.4 N·mm^−1^, respectively, while wet skid resistance is preserved and both rolling resistance and wear resistance are further improved. The findings of this work establish a practical and efficient strategy for the wet preparation of high-performance NR/silica composites.

## 1. Introduction

With the continued advancement of the “green tire” concept [1], the development of high-performance rubber/silica composites that simultaneously exhibit low rolling resistance and excellent wet skid resistance has become a central objective of the tire industry. Silica has been widely adopted as a reinforcing filler owing to its ability to effectively reduce hysteresis loss [2,3]. Nevertheless, the high density of silanol groups (Si-OH) on the silica surface promotes strong interparticle hydrogen bonding, leading to pronounced self-aggregation [4]. As a result, conventional dry mixing processes often suffer from poor filler dispersion, high energy consumption, and severe dust pollution [5]. To overcome these limitations, latex compounding technology has been proposed and progressively developed [6,7]. This technology employs the inherent properties of NR, which is present in the form of aqueous latex [8,9], to achieve an in situ composite of filler and rubber particles in the liquid phase. The process offers dual advantages in terms of energy saving, environmental protection and good dispersibility, and is regarded as the most promising process route for preparing NR/silica composites [10]. However, in actual production, as the amount of silica increases, its loss problem becomes particularly prominent. This phenomenon originates from the tendency of silica particles to self-aggregate un-der high loading conditions [11], which reduces their interaction with rubber latex particles. In the subsequent latex flocculation process, the free aggregates are unable to be effectively wrapped by the rubber phase, and thus are lost with the aqueous phase. This results in a decrease in filler utilisation, deviation of the masterbatch composition from the design formula, and increased manufacturing costs, which seriously restricts the application of latex compounding in high filler systems [12,13,14]. Previous studies have attempted to mitigate silica loss by modifying the surface of the silica [6,13,15] or by introducing surfactants [16] to improve dispersion stability in aqueous media. However, these methods are often accompanied by increased process complexity, elevated costs or higher energy consumption. Therefore, developing a simple and efficient strategy to minimize silica loss during latex compounding remains a critical research priority.

In recent years, clay mineral materials with special structural characteristics [17,18,19] have gradually attracted attention and are considered to play a unique role in rubber composite systems. Among them, sepiolite is a natural magnesium-rich hydrated silicate clay mineral with a special one-dimensional fibrous crystal morphology [20]. The prevailing hypothesis concerning the crystal lattice of the substance under consideration is that it consists of Si-O tetrahedral plates and Mg-O octahedral plates [21]. In addition, it has been determined that the substance exhibits a 2:1 type banded structure parallel to the C-axis. The octahedral plates in sepiolite crystals are characterised by discontinuity. These plates are connected to the anti-tetrahedral plates by alternating bonds formed by oxygen atoms, forming a large number of pore structures [22,23] and abundant silanol groups (Si-OH) on the surface [24,25]. The fibrous structure of sepiolite, combined with its high specific surface area and abundant active silanol groups, renders it effective across diverse fields—including adsorption [26,27,28], catalysis [29], polymer reinforcement [30,31,32], and the stabilization of aqueous suspensions [24,33]. Nevertheless, native sepiolite fibers tend to aggregate into bundled structures as a result of hydrogen bonding and van der Waals interactions. In addition, the complex geological formation conditions of sepiolite deposits often lead to the coexistence of accessory minerals [20,34], such as calcite, quartz, and talc. These factors collectively reduce the effective surface area and surface activity of sepiolite, thereby limiting its application as a nanostructured material [24]. Previous studies [35] have demonstrated that depolymerization and activation treatments can effectively disassemble sepiolite aggregates, enabling the formation of stable three-dimensional networks in liquid media through physical interlocking and weak intermolecular interactions [36]. Such networks not only allow long-term self-suspension of sepiolite but also exert a spatial confinement effect on inorganic particles [37], even in the absence of surfactants, thereby restricting particle migration, sedimentation, and self-aggregation. For example, Fernandes et al. [38] achieved stable aqueous dispersion of carbon nanotubes using sepiolite under ultrasonic treatment without surfactants or oxidative modification, leading to the fabrication of freestanding buckypaper. Similarly, Ruiz-Hitzky and co-workers reported the surfactant-free stabilization of graphene [39], halloysite, and kaolinite [40] via ultrasonic-assisted sepiolite dispersion, enabling the preparation of conductive and separation membranes that combine rigidity with flexibility. Collectively, these findings provide important insights into the use of sepiolite for regulating filler dispersion in wet-processed rubber composite systems.

In rubber materials research, sepiolite has been utilized as a reinforcing filler in NR [41,42], epoxidized natural rubber [43] and various synthetic rubber systems [44,45], exhibiting promising reinforcing effects. Previous studies [35,46,47] indicate that NR molecular chains, as well as non-rubber components such as phospholipids and proteins located at the chain ends, can be adsorbed onto the surface of sepiolite. This adsorption promotes the formation of strong interfacial interactions between sepiolite and the rubber matrix, thereby providing a structural basis for the development of high-performance NR composites. Closely related to this, it has been reported that sepiolite can interact with zero- or quasi-zero-dimensional fillers, such as silica, to construct multiscale synergistic filler networks [48,49,50,51], which under appropriate conditions further enhance the dynamic and static mechanical properties of rubber composites. Notably, existing studies of such multiscale filler systems have predominantly focused on dry mixing processes, whereas the role of sepiolite in regulating silica dispersion, suppressing filler loss, and influencing composite performance during wet latex compounding remains insufficiently explored.

Against this background, the present work proposes the use of disaggregated and activated sepiolite exhibiting a spatial confinement effect as both a suspension stabilizer and a synergistic reinforcing phase to fabricate NR/silica/sepiolite composites by latex compounding method. The specific objectives of this study are: (1) To determine the rheological percolation threshold of disaggregated sepiolite in aqueous media and establish its correlation with suspension stabilization capacity; (2) To systematically investigate the effect of sepiolite on suppressing silica loss during latex compounding; (3) To evaluate the influence of sepiolite incorporation on the dynamic and static mechanical properties of NR/silica composites. The novelty of this investigation lies in: (1) The first application of sepiolite as a dual-functional additive (suspension stabilizer and reinforcing filler) in the wet latex compounding process; (2) The demonstration that sepiolite can effectively suppress silica self-aggregation and reduce filler loss without requiring complex surface modification or surfactants; (3) The establishment of a multiscale synergistic filler network that simultaneously improves tensile strength, tear strength, rolling resistance, and wear resistance while maintaining wet skid resistance.

These suspensions were subsequently incorporated into natural rubber latex through latex compounding to obtain the corresponding composites. By combining direct observation with quantitative analysis, the influence of sepiolite loading on suspension stability and silica loss behavior during wet compounding was systematically evaluated, and the underlying mechanisms were discussed. In parallel, the effects of sepiolite on filler dispersion as well as on the dynamic and static mechanical properties of the composites were examined. The results demonstrate that the introduction of an appropriate amount of sepiolite into the wet latex system effectively suppresses silica loss without relying on complex surface modification, while enabling the construction of a multiscale synergistic filler network within the rubber matrix, thereby achieving both process optimization and performance enhancement.

## 2. Materials and Methods

### 2.1. Materials

The materials used in this study are listed in Table 1.

### 2.2. Preparation of Sepiolite Suspensions with Different Solid Contents

Activated sepiolite powder with a high specific surface area (BET ≥ 235 m^2^/g) was first obtained via ultrasonic disaggregation followed by oxalic acid activation [35]. The treated sepiolite was then dispersed in deionized water to prepare suspensions with solid contents of 0.1%, 0.2%, 0.3%, 0.4%, 0.5%, 0.6%, 0.7%, 0.8%, 0.9%, 1.0%, 1.2%, 1.4%, 1.6%, 1.8%, 2.0%, 2.5%, 3.0%, 3.5%, 4.0%, 4.5%, and 5.0% (*w*/*w*). Each suspension was subjected to ultrasonic dispersion for 15 min, after which its viscosity was measured. Subsequently, the suspensions were transferred into 50 mL glass vials and allowed to stand under quiescent conditions for visual observation.

### 2.3. Preparation of Silica/Sepiolite Slurries

Silica and sepiolite were weighed according to dry mass ratios of 50:0, 49:1, 48:2, 47:3, 46:4, 45:5, 44:6, 43:7, 42:8, 41:9, and 40:10, respectively. The solids were added to deionized water to obtain aqueous slurries with a total solid content of 10 wt%. The mixtures were stirred at 1000 rpm for 30 min and subsequently subjected to ultrasonic dispersion for 15 min to yield homogeneous silica/sepiolite suspensions. By maintaining a constant total solid content of 10 wt%, the sepiolite fraction in the aqueous slurries corresponded to 0%, 0.2%, 0.4%, 0.6%, 0.8%, 1.0%, 1.2%, 1.4%, 1.6%, 1.8%, and 2.0%, respectively. Portions of each slurry were transferred into 50 mL glass vials and stored under static conditions for observation. The samples were designated as H50S0, H49S1, H48S2, H47S3, H46S4, H45S5, H44S6, H43S7, H42S8, H41S9, and H40S10 based on the dry mass ratio of silica to sepiolite.

### 2.4. Preparation of NR/Silica Composites

The concentration of NRL was first adjusted to 30 wt%. According to the formulation listed in Table 2, the diluted latex was gradually introduced into the silica slurry and mechanically stirred at 500 rpm for 30 min to ensure homogeneous mixing. Coagulation was subsequently induced by the dropwise addition of formic acid until no further rubber flocs were observed. The resulting masterbatch was repeatedly soaked and washed with deionized water to remove residual impurities, followed by drying in a forced-air convection oven. Meanwhile, the supernatant remaining after coagulation was allowed to stand in a beaker, and the solid residue obtained after drying was collected to evaluate filler loss during the latex compounding process.

Prior to compounding, the dried NR masterbatch was passed ten times through an open two-roll mill to achieve uniform plasticization. TESPT, ZnO, stearic acid, accelerators CBS and DM, and S were then sequentially incorporated, and the total mixing time was controlled at 15 min. Vulcanization was carried out in a hydraulic hot press with the temperature and pressure set to 150 °C and 10 MPa, respectively. The curing time was determined as t_90_ + 3 min based on measurements obtained from moving die rheometer (MDR). The resulting vulcanizates were designated as NR/H50S0, NR/H48S2, NR/H46S4, NR/H44S6, NR/H42S8, and NR/H40S10, respectively. The processing parameters, including ultrasonic dispersion time, stirring speed, and curing conditions, were selected based on preliminary optimization experiments and references to prior studies [35,46]. Although a formal design-of-experiments (DOE) approach was not employed, the chosen conditions ensured consistent and reproducible composite fabrication.

### 2.5. Characterizations

The viscosity of the sepiolite suspensions was measured at 25 °C using an NDJ-9S digital rotational viscometer (manufactured by Shanghai Fangrui Instrument Co., Ltd. Shanghai, China), with a measurement accuracy of ±1%. Temperature control was achieved using a CH1006N thermostatic water bath integrated with the viscometer, providing a temperature stability of ±0.1 °C.

The filler content in the NR composites was determined by thermogravimetric analysis using a STA 449 (F5) thermal analyzer (NETZSCH-Gerätebau GmbH, Selb, Germany). Approximately 5 mg of each sample was placed in a platinum crucible and heated from room temperature to 700 °C at a heating rate of 10 °C min^−1^ under an air atmosphere.

The Payne effect of the rubber composites was evaluated using a rubber process analyzer (RPA2000, Alpha Technologies, Akron, OH, USA). Strain sweep tests were conducted at 60 °C and 1 Hz, with the strain amplitude ranging from 0.28% to 100%, and the corresponding storage modulus was recorded.

The microstructural morphology of the cryo-fractured surfaces of the vulcanized composites were examined using a field-emission scanning electron microscope (JSM-7500F, JEOL, Tokyo, Japan). All samples were sputtered with gold before observations. The presented SEM images are representative micrographs used for qualitative comparison of filler dispersion and interfacial morphology.

Cure characteristics were measured using a moving die rheometer (MDR2000, Alpha Technologies, Akron, OH, USA) at 150 °C, and the test duration was set to 30 min.

The mechanical properties of the vulcanized composites were measured using a universal testing machine (Z020, Zwick/Roell GmbH Co., Ulm, Germany). Tensile strength and tear strength were determined according to ISO 37:2024 [52] and ISO 34-1:2022 [53], respectively. Type 2 dumbbell-shaped specimens were used for tensile testing, while right-angle specimens were used for tear testing. All tests were performed at a crosshead speed of 500 mm min^−1^. Five samples were tested for each formulation, and the median value was reported.

Dynamic mechanical properties were evaluated using a DMA Q800 analyzer (TA Instruments, New Castle, DE, USA). Temperature sweep tests were conducted from −80 °C to 80 °C at a heating rate of 3 °C min^−1^, with a strain amplitude of 0.1% and a frequency of 10 Hz.

Abrasion resistance was determined using a DIN abrasion tester (GT-7012-D, GOTECH Testing machines Co., Ltd., Taiwan, China). Prior to testing, debris on the rotating drum was removed and the initial mass of each specimen (m_1_) was recorded. The specimen was fixed in the holder with an exposed length of approximately 2 mm. After testing, residual debris was removed and the final mass (m_2_) was measured. The abrasion volume was calculated based on the mass loss and the density (ρ) of the rubber composite, determined in accordance with ISO 4649-2024 [54].

## 3. Results and Discussion

### 3.1. Stability of Sepiolite Suspensions with Different Solid Contents

Previous studies have demonstrated that the rheological percolation threshold of sepiolite in aqueous media plays a decisive role in its ability to stabilize other dispersed inorganic phases. For example, Fernandes et al. [38] determined the percolation threshold of a commercial rheological-grade sepiolite (Pangel) by analyzing the concentration dependence of rotational viscosity, and subsequently compared its effectiveness in stabilizing carbon nanotubes below and above this threshold. Along similar lines, Ruiz-Hitzky et al. [40] employed the same rheological criterion to evaluate the suspension-stabilizing capability of sepiolite toward kaolinite and halloysite nanotubes at different concentrations. These studies indicate that identifying the percolation threshold of sepiolite in water is a prerequisite for rationally designing sepiolite-based suspension systems for inorganic fillers. Therefore, the viscosity evolution and suspension characteristics of the disaggregated and activated sepiolite at various solid contents were investigated using rotational viscometry combined with direct visual observation, aiming to provide application guidance for the subsequent stabilization of silica slurries.

**Figure 1:**
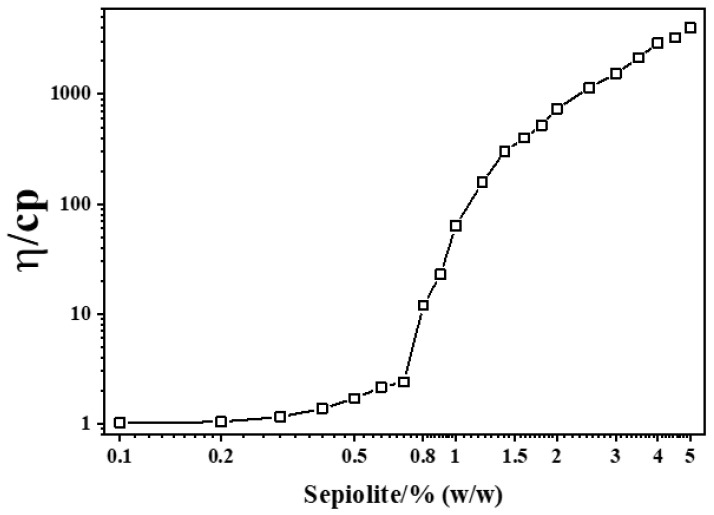
Viscosity data of different solid content sepiolite aqueous phase suspensions.

**Figure 2:**
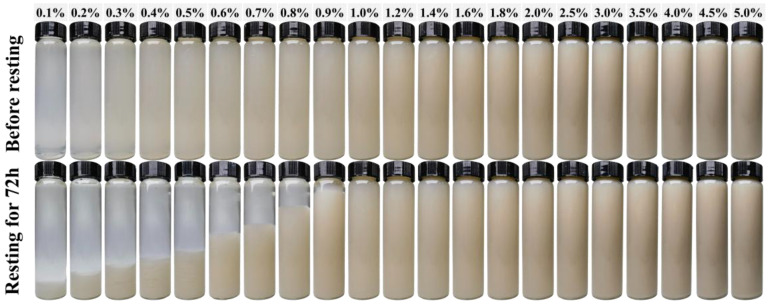
State diagram of different solid content sepiolite aqueous phase suspensions before and after resting.

Figure 1 presents the viscosity data of different solid content sepiolite aqueous phase suspensions. At concentrations below 0.7 wt%, the viscosity exhibited only minimal variation. However, when the solid content exceeded 0.8%, the viscosity increased nearly linearly with increasing concentration. This transition suggests that the rheological percolation threshold of the disaggregated and activated sepiolite used in this study is located in the vicinity of 0.8 wt%. This conclusion is further corroborated by static sedimentation observations (Figure 2), where suspensions containing ≥1.0 wt% sepiolite exhibited complete stability after 72 h, while those below 0.8 wt% showed progressive sedimentation. The intermediate range (0.8–1.0 wt%) displayed partial stabilization, consistent with the formation of an incipient percolating network. Notably, this value is higher than the percolation threshold of approximately 0.2 wt% reported by Fernandes et al., a discrepancy that can reasonably be attributed to differences in mineral composition, fiber morphology, and surface activity of sepiolite from different sources [38].

The suspension stability of sepiolite dispersions before and after static aging for 72 h was further examined, as shown in Figure 2. Immediately after ultrasonic disaggregation, all suspensions exhibited a homogeneous appearance, irrespective of solid content. After resting for 72 h, the stability of the suspensions increased progressively with increasing sepiolite concentration, albeit with notable non-uniform variations in the intermediate concentration range (0.8–1.0 wt%). This non-uniform behavior is attributed to the percolation threshold effect: below 0.8 wt%, the fiber concentration is insufficient to form a continuous network; at the threshold, small concentration increases induce significant structural changes; and above 1.0 wt%, the fully percolated network provides uniform stabilization. This phenomenon is closely related to the structural characteristics and morphological evolution of sepiolite fibers. Upon ultrasonic treatment, sepiolite bundles were progressively separated into individual rod-like nanofibers. During the resting process, these fibers became interconnected through physical entanglement, giving rise to a continuous three-dimensional network structure. Concurrently, the internal channels and surface silanol groups of sepiolite facilitated hydrogen bonding interactions with free water molecules [55]. When the sepiolite concentration is low, the formed three-dimensional network structure is limited, and the system exhibits poor suspension stability within a certain volume. However, as the concentration increases, the network structure gradually becomes more complete and covers the entire volume, and the system exhibits a stable suspension state. These provide a theoretical and practical foundation for the stabilization of silica slurries using sepiolite suspensions.

### 3.2. Effect of Sepiolite on the Wet Preparation of NR/Silica Masterbatches

#### 3.2.1. Influence of Sepiolite on the Suspension Stability of Silica Slurries

**Figure 3:**
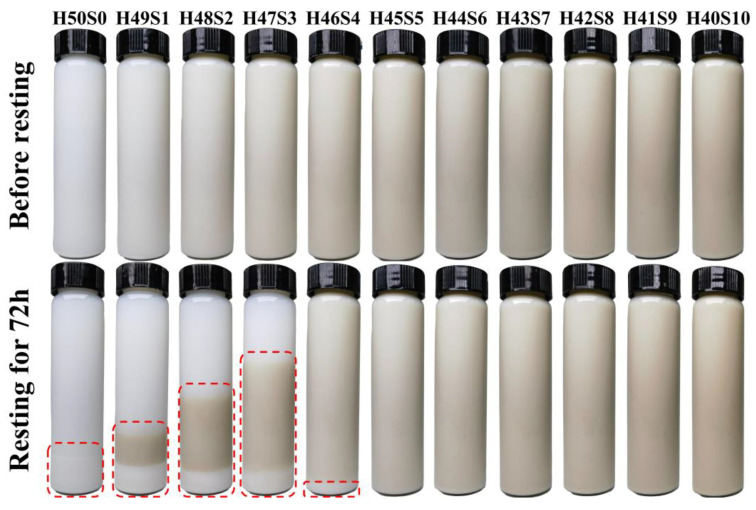
State diagram of 10% silica/sepiolite aqueous dispersions before and after resting (Sepiolite concentrations from left to right are 0%, 0.2%, 0.4%, 0.6%, 0.8%, 1.0%, 1.2%, 1.4%, 1.6%, 1.8%, 2.0%).

Figure 3 illustrates the stability of silica/sepiolite aqueous slurries with a total solid content of 10% before and after resting for 72 h. In the absence of sepiolite (H50S0), pronounced sedimentation of silica was observed after aging, indicating poor suspension stability. When the sepiolite content was lower than 0.8 wt% (H49S1, H48S2, H47S3, and H46S4), the suspensions exhibited clear stratification. In these samples, the bottom layer corresponded to settled silica, while the upper layer contained only a small amount of suspended silica, similar to that observed in H50S0. Based on the compositional ratios of silica and sepiolite, as well as the relative heights of the sedimented and intermediate layers, the middle layer can be reasonably assigned to a mixed suspension of sepiolite and silica. With increasing sepiolite content, the volume fraction of both the bottom sediment and the upper dilute layer gradually decreased, whereas the intermediate mixed suspension layer became more prominent. At a sepiolite content of 0.8 wt% (H46S4), only a minor amount of silica sediment was detected after 72 h, while the majority of the slurry remained homogeneously suspended. When the sepiolite content exceeded 1.0 wt% (H45S5 to H40S10), no discernible sedimentation or phase separation was observed after resting, and the slurries retained good fluidity. These results demonstrate that the sepiolite content above 1.0 wt% is sufficient to ensure long-term suspension stability of silica slurries. Subsequently, NR/silica/sepiolite composites were prepared by mixing the selected silica/sepiolite suspension with NRL using the latex compounding method.

#### 3.2.2. Silica Loss During Latex Compounding Process

**Figure 4:**
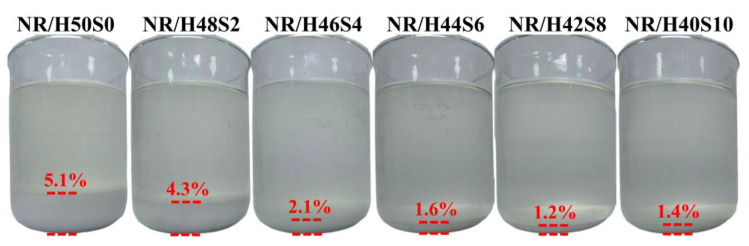
The diagram of comparison for the remaining flocculant after latex compounding.

In order to evaluate the influence of sepiolite addition on silica retention, the residual solids in the flocculation serum and the thermogravimetric behavior of the NR/silica masterbatches were analyzed. Figure 4 presents photographs of the collected flocculation serum after standing for 48 h. The percentage values represent the mass ratio of the dried residue to the total dry mass of the NR, silica and sepiolite added at the beginning. A pronounced loss was observed for the NR/H50S0 sample, with a flocculation loss rate of up to 5.1%. As the sepiolite content increased, the loss rate exhibited a monotonic decrease. In particular, for the NR/H42S8 sample, the loss rate was reduced to 1.2%, indicating that the incorporation of an appropriate amount of sepiolite effectively suppresses filler loss during wet latex compounding.

**Figure 5:**
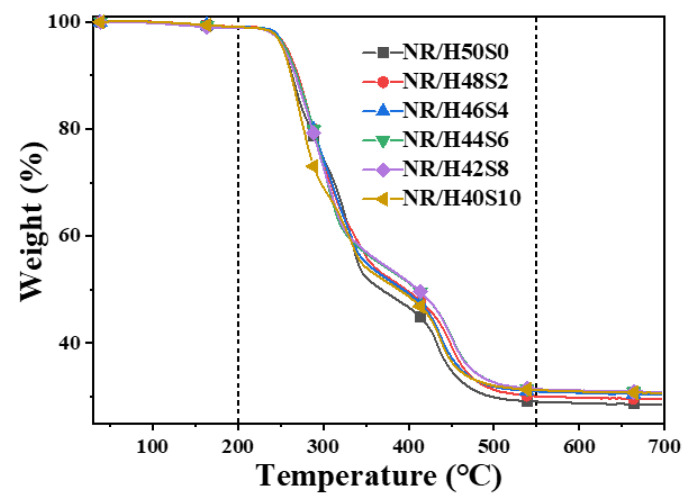
TG curves of the compound masterbatch with different amount of sepiolite.

To further quantify the effect of sepiolite content on filler retention, thermogravimetric analysis (TGA) was performed on the obtained masterbatches, as shown in Figure 5. Below 200 °C, all samples exhibited comparable and minimal mass loss, which can be attributed primarily to the evaporation of residual moisture. In the temperature range of 200–550 °C, noticeable differences in mass loss were observed, corresponding predominantly to the thermal degradation of natural rubber. Above 550 °C, the residual mass of all samples converged to similar values.

On this basis, the mass loss in the range of 200–550 °C was assumed to originate exclusively from NR decomposition. The filler loss during wet compounding was therefore estimated according to Equation (1).

(1) $$\mathrm{Loss}\;\mathrm{rate}=\left(1-\frac{M_{\mathrm{NR}}}{W_{200–550}\left(M_{\mathrm{NR}}+M_{s}\right)}\right)\times 100\%$$

The calculated results, summarized in Table 3, reveal that samples NR/H50S0 and NR/H48S2 contained higher proportions of NR and correspondingly higher silica loss rates of 4.7% and 3.4%, respectively. With increasing sepiolite content, the silica loss rate decreased markedly. For NR/H44S6 and NR/H42S8, corresponding to sepiolite contents of 1.2% and 1.6% in the slurry, the silica loss rate was reduced to 1.4% and 1.1%, respectively, in good agreement with the flocculation residue analysis. These results confirm that sepiolite addition effectively mitigates filler loss during wet latex compounding, thereby improving masterbatch compositional fidelity. The reduction in filler loss from 4.7% to 1.1% (a 76.6% decrease) is attributed to the synergistic effects of: (1) three-dimensional network formation by sepiolite fibers that physically constrains silica particles and prevents aggregate formation prior to coagulation; (2) enhanced rubber-filler interfacial interactions facilitated by sepiolite’s silanol groups and surface activity; and (3) modified coagulation kinetics that promote more effective encapsulation of well-dispersed silica particles by the coagulating rubber phase.

#### 3.2.3. Mechanism of Sepiolite-Assisted Silica Stabilization and Reduction in Filler Loss

**Figure 6:**
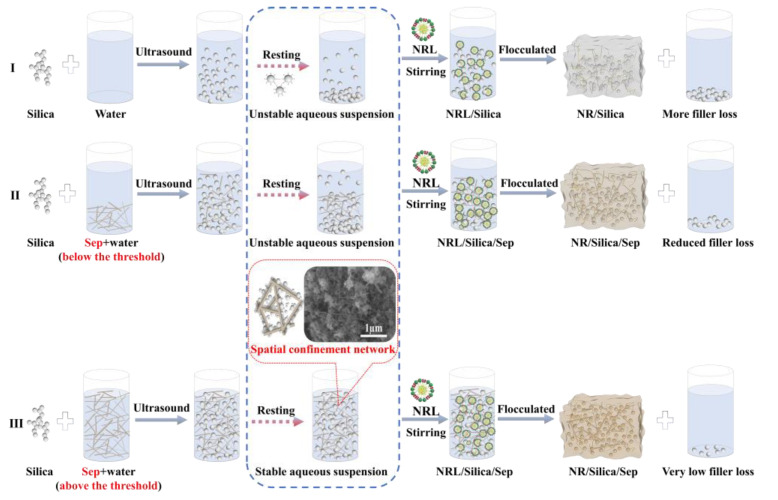
Schematic diagram of sepiolite suspension stabilized silica and the improvement mechanism of latex compounding process. (I) Preparation of NR/Silica composites; (II) Preparation of NR/Silica/minor sepiolite composites; (III) Preparation of NR/Silica/an appropriate dosage of sepiolite composites.

Based on the experimental results discussed above, a schematic illustration describing the role of sepiolite in stabilizing silica suspensions and mitigating filler loss during latex compounding is proposed, as shown in Figure 6. In the absence of sepiolite, silica particles at relatively high concentrations readily undergo self-aggregation through hydrogen bonding between surface silanol groups. As a consequence, their collision frequency with natural rubber latex particles is reduced. During latex destabilization, a fraction of silica particles therefore fails to establish effective contact with, or become encapsulated by, rubber molecular chains, resulting in filler loss. This loss becomes more pronounced with increasing silica loading. When the sepiolite content in the aqueous dispersion exceeds its rheological percolation threshold, a sufficient number of fibers is present to form an interconnected three-dimensional network through physical entanglement, assisted by weak interfacial interactions. Upon incorporation of silica, the particles become confined within this fibrous network and are thus prevented from forming large, sedimentable aggregates. In this context, the sepiolite network imposes a spatial confinement effect that stabilizes the silica slurry. Following the introduction of NRL, the effective contact probability between silica and rubber particles is substantially increased. During flocculation, rubber molecular chains are more uniformly adsorbed, wrapped, or entangled around the filler particles through the combined adsorption of sepiolite and silica, leading to the formation of a homogeneous and stable masterbatch with reduced filler loss. By contrast, when the sepiolite content remains below the percolation threshold, the resulting network is insufficiently developed to restrict silica aggregation and sedimentation, thereby limiting filler–latex interactions and giving rise to measurable filler loss.

### 3.3. Effect of Sepiolite on the Properties of NR/Silica Composites Prepared by Latex Compounding

#### 3.3.1. Filler Dispersion and Interfacial Interactions at Different Sepiolite Loadings

**Figure 7:**
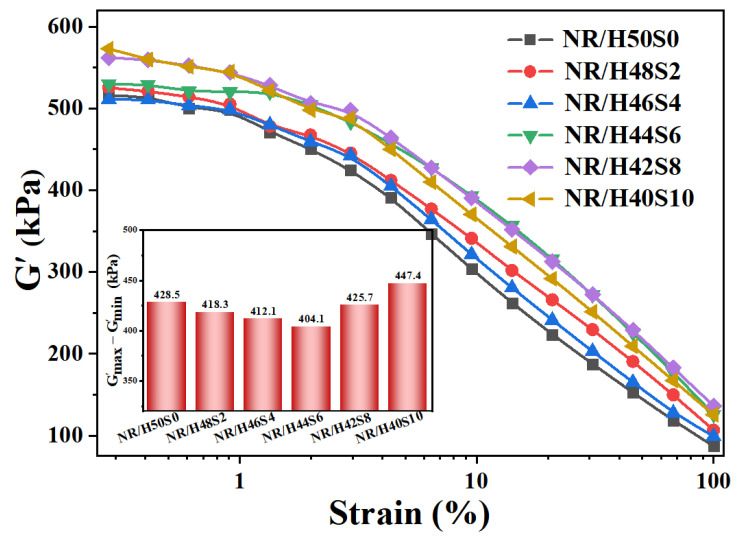
The storage modulus (G′)-strain curve of the uncured composites with different amount of sepiolite.

As shown in Figure 7, the Payne effect parameter (ΔG′) of the uncured NR/silica compounds exhibits a non-monotonic dependence on sepiolite content, decreasing initially and subsequently increasing with further addition. Compared with the sepiolite-free compound (NR/H50S0), a pronounced reduction in ΔG′ is observed for NR/H46S4 and NR/H44S6, indicating that an appropriate amount of sepiolite effectively suppresses filler–filler aggregation. This behavior is primarily attributed to the wet-emulsion compounding process, during which sepiolite provides effective suspension stabilization for silica particles, thereby increasing their probability of contact with the rubber latex. As a consequence, silica particles are more uniformly dispersed throughout the rubber matrix, leading to a weakened filler network. When the sepiolite loading is further increased, ΔG′ rises again. This increase is associated with a gradual transition in the dominant filler network structure—from one governed mainly by silica–silica interactions to one dominated by interactions between sepiolite nanofibers and silica particles, as well as inter-fiber entanglements. The formation of a reinforced hybrid filler network enhances the elastic response of the compound, resulting in an increased ΔG′ value.

**Figure 8:**
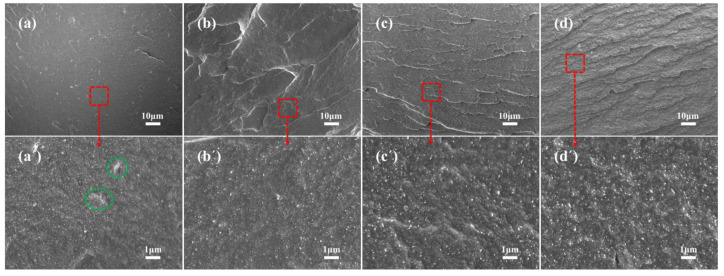
SEM images of NR/silica composites: (**a**,**a’**) NR/H50S0; (**b**,**b’**) NR/H46S4; (**c**,**c’**) NR/H42S8; (**d**,**d’**) NR/H40S10.

Figure 8 presents representative SEM micrographs of the cryo-fractured surfaces of NR/silica composites with varying sepiolite contents. In the control sample NR/H50S0 (without sepiolite), numerous large silica aggregates with sizes exceeding 1 μm are clearly visible on the fracture surface (indicated by red arrows in Figure 8a). These aggregates exhibit smooth surfaces and sharp boundaries with the rubber matrix, suggesting weak interfacial adhesion. The presence of micro-voids between aggregates and matrix further indicates poor stress transfer efficiency. With the incorporation of sepiolite (NR/H46S4, Figure 8b), the average size of silica aggregates decreases noticeably, and the distribution becomes more uniform. Sepiolite fibers are identifiable as needle-like structures bridging between adjacent silica particles (indicated by yellow arrows). These fibers appear to penetrate into both silica aggregates and the rubber matrix, suggesting effective interfacial bonding. At the optimal sepiolite loading (NR/H42S8, Figure 8c), the fracture surface exhibits the most homogeneous morphology with minimal visible aggregates. The sepiolite fibers are well-dispersed and appear to form an interconnected network that encapsulates silica particles. The rubber matrix shows substantial plastic deformation around the filler structures, indicating effective stress transfer under mechanical loading. This morphology is consistent with the observed improvements in tensile and tear strengths. Further increasing sepiolite content to 10 phr (NR/H40S10, Figure 8d) results in the appearance of some sepiolite fiber bundles and a rougher fracture surface. While the filler dispersion remains acceptable, the presence of these bundles suggests approaching the threshold for optimal filler distribution, consistent with the mechanical property trends shown in Figure 7. Although the SEM images shown are representative rather than statistically analyzed datasets, their observations are consistent with the Payne effect results and mechanical property variations, collectively supporting improved filler dispersion in the presence of sepiolite.

#### 3.3.2. Cure Characteristics of NR/Silica Composites at Different Sepiolite Loadings

The vulcanization characteristics of rubber composites can be utilised to evaluate the reactivity, flowability, and the strength of the cross-linked network structure during the transition from a two-dimensional linear structure to a three-dimensional cross-linked network structure. The curing parameters of NR/silica composites containing different amounts of sepiolite are illustrated in Table 4. It is observed that both the scorch time (t_10_) and optimum cure time (t_90_) increase progressively with increasing sepiolite content. This trend is attributed to the large specific surface area and high surface activity of sepiolite, which promote the adsorption of curatives and accelerators more than silica, thereby retarding the vulcanization process.

The minimum torque (ML), reflecting both the flow behavior of the uncured compound and the degree of filler networking, exhibits an initial decrease followed by an increase with rising sepiolite content. This variation is consistent with the trend observed for the Payne effect, suggesting a similar evolution of the filler network structure. Meanwhile, the torque difference (MH-ML) increases monotonically as the sepiolite loading increases, indicating a progressive enhancement of the crosslinking network. This enhancement is associated with the fibrous morphology of sepiolite, which facilitates stronger physical adsorption and chain entanglement with the rubber matrix, thereby leading to a denser and more robust network structure.

#### 3.3.3. Dynamic and Static Dynamic Mechanical Properties of NR/Silica Composites at Different Sepiolite Loadings

**Figure 9:**
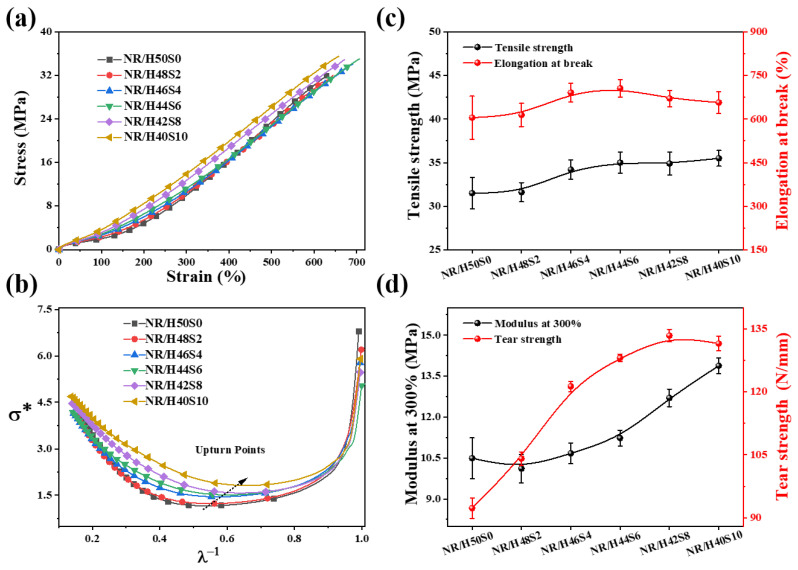
(**a**) Stress–strain curves; (**b**) Mooney-Rivlin fitted curves; (**c**) Tensile strength and elongation at break; (**d**) Modulus at 300% and tear strength of the natural rubber/silica composites with different amount of sepiolite.

Figure 9a presents the stress–strain curves of the NR/silica composites containing different amounts of sepiolite. The corresponding mechanical parameters are listed in Table 5, and the variations in tensile strength, elongation at break, modulus at 300% strain, and tear strength are illustrated in Figure 9c,d. As the sepiolite content increases, the tensile strength, modulus at 300% and tear strength exhibit a continuous upward trend. Specifically, the tensile strength increases from 32.1 to 35.5 MPa, while the tear strength rises from 92.3 to 133.4 N·mm^−1^, indicating a pronounced synergistic reinforcement effect. This enhancement is attributed to the improved dispersion of silica within the NR matrix facilitated by the latex compounding process in the presence of sepiolite. In addition, the large specific surface area and high surface activity of sepiolite promote stronger interfacial interactions with the rubber matrix, which favor efficient stress transfer under external loading. With respect to tear resistance, the incorporation of anisotropic sepiolite fibers effectively impedes crack propagation during tearing, thereby leading to a substantial increase in tear strength. In contrast, the elongation at break initially increases and subsequently decreases with increasing sepiolite content. At moderate loadings, sepiolite promotes uniform filler dispersion and homogeneous filler–rubber interactions, resulting in enhanced toughness. At higher loadings, however, the strong interactions between sepiolite and the rubber matrix restrict chain mobility, leading to reduced ductility and increased brittleness.

Based on the stress–strain results, the Mooney–Rivlin model (Equation (2)) was employed to further elucidate the effect of sepiolite incorporation on the interfacial strength between the filler and the rubber matrix. The stress–strain curves shown in Figure 9a were fitted accordingly, yielding the σ*–λ^−1^ plots presented in Figure 9b. This representation is commonly used to evaluate the filler network structure and the interfacial interactions between fillers and the rubber matrix in rubber composites [56,57].

The Mooney–Rivlin equation is expressed as follows:

(2) $$\sigma^{*}=\frac{\sigma }{\lambda -\lambda^{-2}}=2C_{1}+2C_{2}\times \lambda^{-1}$$

where σ is the tensile stress, λ is the corresponding stretch ratio, the C_1_ and C_2_ are constants independent of λ. The Mooney–Rivlin framework is widely applied to describe changes in the filler–rubber network structure in filled elastomer systems.

At relatively low strains (corresponding to higher λ^−1^ values), σ* decreases sharply, which can be attributed to the progressive breakdown of the filler network under tensile deformation. With increasing sepiolite content, the strain corresponding to the onset of the σ* upturn (denoted as the upturn point) gradually shifts to lower deformation levels, as reflected by larger λ^−1^ values. This upturn point is generally considered indicative of the interfacial bonding strength between the rubber matrix and the filler. Stronger interfacial interactions result in a higher fraction of rubber chains being anchored to the filler surface, thereby restricting chain mobility and leading to the appearance of the upturn at lower strains. These results collectively indicate that the filler–rubber interactions are progressively enhanced with increasing sepiolite content.

The mechanical enhancement mechanism of sepiolite on NR/silica composites was comprehensively substantiated by the combination of macroscopic mechanical property characterization, microscopic morphological observation, rheological analysis, and Mooney-Rivlin model fitting, and the multiple characterization results form a mutually corroborative mechanistic evidence chain: (1) The enhanced filler-rubber interfacial interactions (quantitatively reflected by the Mooney-Rivlin model) ensure efficient load transfer from the rubber matrix to the rigid filler phase under external loading; (2) The anisotropic sepiolite nanofibers act as a “bridge” at the crack tip (directly observed by SEM rough fracture surfaces), hindering crack propagation and causing crack deflection/pinning, which consumes more fracture energy and improves tear strength; (3) The sepiolite 3D network exerts a spatial confinement effect on silica particles, and the uniform dispersion of silica and sepiolite constructs a multiscale synergistic filler network (zero-dimensional silica + one-dimensional sepiolite) in the rubber matrix, which further improves the load-bearing capacity and stress transfer efficiency of the composite.

**Figure 10:**
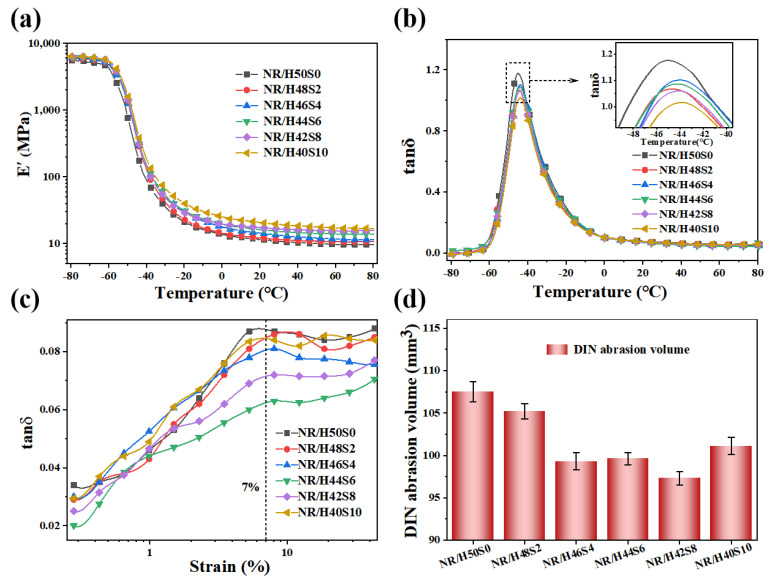
Temperature dependence of (**a**) the storage modulus (E′) and (**b**) the loss factor (tan δ); (**c**) The loss factor (tan δ)-strain curve and (**d**) DIN abrasion volume of the cured NR/silica composites with different amount of sepiolite.

Figure 10a presents the temperature dependence of the E′ for the composites. Compared with NR/H50S0, a gradual increase in E′ is observed with increasing sepiolite content. At 25 °C, the E′ value of NR/H40S10 reaches 20.2 MPa, representing an increase of 79.4% relative to NR/H50S0, indicating that sepiolite incorporation enhances the interfacial interactions between the rubber matrix and the fillers. Figure 10b shows the temperature dependence of the tan δ, with the corresponding values summarized in Table 6. As shown, all composites exhibit a pronounced tan δ peak at approximately −45 °C, which is generally associated with the glass transition temperature (Tg) of the vulcanizates. Upon the addition of sepiolite, Tg shifts toward higher temperatures, suggesting an increased restriction of rubber chain mobility due to strengthened filler–rubber interactions.

With respect to tire tread applications, silica-reinforced NR composites are typically evaluated based on tan δ at 0 °C and 60 °C, which are commonly correlated with wet skid resistance and rolling resistance, respectively. The tan δ values at 0 °C remain nearly unchanged with increasing sepiolite content. In contrast, tan δ at 60 °C initially decreases and then increases; however, this trend cannot fully reflect rolling resistance under practical conditions, as the filler network remains largely intact under small-strain testing. Therefore, the strain-dependent tan δ behavior at 60 °C was further examined at higher strain levels (7%), as shown in Figure 10c. The results indicate a similar trend, with the minimum tan δ observed for NR/H44S6, suggesting that an appropriate sepiolite content promotes filler dispersion and interfacial bonding while reducing frictional energy dissipation between fillers. At higher sepiolite contents, increased friction associated with sepiolite–sepiolite and sepiolite–silica interactions lead to a rise in energy loss. Figure 10d illustrates the abrasion volume as a function of sepiolite content. The abrasion volume decreases initially and then increases with increasing sepiolite loading, reaching a minimum when the sepiolite fraction accounts for 8–16% of the total filler content. These results indicate that, within an appropriate range, sepiolite incorporation in the wet compounding process improves rolling resistance and abrasion resistance without compromising wet skid performance.

## 4. Conclusions

In this study, disaggregation-activated sepiolite was introduced as a suspension stabilizer in latex compounding systems to fabricate NR/silica composites with varying sepiolite contents. The role of sepiolite in regulating filler stability, interfacial structure, and composite performance was systematically investigated. Rheological analysis demonstrated that when the sepiolite solid content reached ≥1.0 wt%, the three-dimensional fibrous network constructed in the aqueous phase generated a pronounced spatial confinement effect, which effectively suppressed silica sedimentation and agglomeration. As a consequence, the silica loss during latex compounding decreased from approximately 4.7% to 1.1% (a 76.6% reduction), indicating improvement in filler utilization efficiency. Although the introduction of sepiolite slightly delayed vulcanization process, the rheological behavior, morphological observations, and mechanical characterization indicated that an appropriate amount of sepiolite can improve the dispersion state of silica within the rubber matrix, and through its fiber-bridging function, sepiolite cooperates with silica to establish a multiscale filler network, thereby enhancing filler–rubber interfacial interaction. Correspondingly, the optimal composite (NR/H42S8 with 8 phr sepiolite) exhibited a 10.6% improvement in tensile strength (from 32.1 MPa to 35.5 MPa), and a 44.5% improvement in tear strength (from 92.3 to 133.4 N·mm^−1^), showing a significant synergistic reinforcing effect. Dynamic mechanical analysis further revealed enhanced interfacial interactions in the presence of sepiolite. Importantly, appropriate sepiolite incorporation enabled simultaneous improvement in rolling resistance and abrasion resistance without compromising wet skid resistance. In summary, sepiolite enables effective control over silica loss and dispersion through physical network regulation, eliminating the necessity for complex surface modification. This approach offers a straightforward, efficient, and sustainable route for the environmentally friendly preparation of high-performance NR/silica composites. Furthermore, this composite exhibits high mechanical strength, low rolling resistance, excellent wet skid resistance and wear resistance, which is especially suitable for green tire tread materials, high-performance rubber damping parts and transmission belt materials, and has broad industrial application prospects in the field of green rubber products.

## Figures and Tables

**Table 1:** Raw materials and their specifications.

Material	Supplier	Specification	Limiting Value
Natural rubber latex (NRL)	Chinese Academy of Tropical Agricultural Sciences, Haikou, China.	Solid content	60%
Precipitated silica (HM-1170)	Jiangxi Black Cat Carbon Black Co., Ltd., Jingdezhen, China.	BET specific surface area	≥160 m^2^/g
Sepiolite	Qingdao Zhongxiang Environmental Protection Technology Co., Ltd., Qingdao, China.	Chemical composition (XRF): 65.19% SiO_2_, 20.25% MgO, 7.89% Al_2_O_3_, 3.11% Fe_2_O_3_, 1.05% CaO	–
Formic acid (HCOOH)	Sinopharm Chemical Reagent Co., Ltd., Shanghai, China.	Analytical grade	≥99.5%
Stearic acid	Sinopharm Chemical Reagent Co., Ltd., Shanghai, China.	Analytical grade	≥98.0%
Zinc oxide (ZnO)	Sinopharm Chemical Reagent Co., Ltd., Shanghai, China.	Analytical grade	≥99.0%
Accelerator CBS	SanLux Co., Ltd., Shaoxing, China.	Industrial grade	≥99.0%
Accelerator DM	SanLux Co., Ltd., Shaoxing, China.	Industrial grade	≥99.0%
Silane coupling agent (TESPT)	SanLux Co., Ltd., Shaoxing, China.	Industrial grade	≥99.0%
Sulfur (S)	SanLux Co., Ltd., Shaoxing, China.	Industrial grade	≥99.0%

**Table 2:** Formulation of NR/silica composites with different sepiolite content/phr.

Samples	Masterbatches			TESPT	ZnO	SA	CBS	DM	S
	NR	HM (SiO_2_)	Sep						
NR/H50S0	100	50	0	4	5	2	2	1	2
NR/H48S2	100	48	2	4	5	2	2	1	2
NR/H46S4	100	46	4	4	5	2	2	1	2
NR/H44S6	100	44	6	4	5	2	2	1	2
NR/H42S8	100	42	8	4	5	2	2	1	2
NR/H40S10	100	40	10	4	5	2	2	1	2

**Table 3:** Loss rate of silica (calculation based on TG data of the compound masterbatch).

Materials	W200–550/%	Remaining/%	Loss Rate/%
NR/H50S0	69.95	29.04	4.7
NR/H48S2	69.06	30.08	3.4
NR/H46S4	68.12	30.95	2.1
NR/H44S6	67.62	31.41	1.4
NR/H42S8	67.42	31.43	1.1
NR/H40S10	67.80	31.25	1.6

**Table 4:** The curing parameters of the NR/silica composites with different amount of sepiolite.

Samples	t_10_/min	t_90_/min	ML/dN∙m	MH/dN∙m	M_H_-M_L_/dN∙m
NR/H50S0	2.65	7.53	2.17	24.23	22.06
NR/H48S2	2.53	7.27	2.13	24.73	22.60
NR/H46S4	2.72	7.90	2.13	25.90	23.77
NR/H44S6	2.92	7.96	2.13	25.65	23.52
NR/H42S8	3.10	8.15	2.15	25.34	23.19
NR/H40S10	3.21	8.03	2.14	26.89	24.75

**Table 5:** The mechanical properties data of the NR/silica composites with different amount of sepiolite.

Samples	Modulus at 100%/MPa	Modulus at 300%/MPa	Tensile Strength/MPa	Elongation at Break/%	Tear Strength/N·mm^−1^
NR/H50S0	2.0 ± 0.3	10.0 ± 0.8	32.1 ± 1.8	633 ± 54	92.3 ± 2.4
NR/H48S2	2.3 ± 0.3	10.3 ± 0.5	32.0 ± 1.1	644 ± 40	104.1 ± 1.6
NR/H46S4	2.6 ± 0.2	10.7 ± 0.4	34.2 ± 1.1	691 ± 33	121.3 ± 1.2
NR/H44S6	2.8 ± 0.1	11.2 ± 0.3	35.0 ± 1.2	706 ± 30	128.1 ± 0.9
NR/H42S8	3.1 ± 0.2	12.7 ± 0.3	34.9 ± 1.3	671 ± 28	133.4 ± 1.5
NR/H40S10	3.6 ± 0.3	13.9 ± 0.6	35.5 ± 1.9	657 ± 37	131.5 ± 1.7

**Table 6:** The dynamic properties data of NR/silica composites with different amount of sepiolite.

Samples	E′ at 25 °C (MPa)	T g (°C)	tan δ at 0 °C	tan δ at 60 °C
NR/H50S0	11.2	−45.1	0.100	0.055
NR/H48S2	11.9	−44.8	0.099	0.056
NR/H46S4	13.9	−44.2	0.100	0.049
NR/H44S6	15.8	−44.4	0.098	0.044
NR/H42S8	17.0	−44.2	0.099	0.051
NR/H40S10	20.2	−44.1	0.101	0.053

## Data Availability

The original contributions presented in this study are included in the article. Further inquiries can be directed to the corresponding authors.
